# Care Gaps and Recommendations in Vestibular Migraine: An Expert Panel Summit

**DOI:** 10.3389/fneur.2021.812678

**Published:** 2022-01-03

**Authors:** Monica P. Mallampalli, Habib G. Rizk, Amir Kheradmand, Shin C. Beh, Mehdi Abouzari, Alaina M. Bassett, James Buskirk, Claire E. J. Ceriani, Matthew G. Crowson, Hamid Djalilian, Joel A. Goebel, Jeffery J. Kuhn, Anne E. Luebke, Marco Mandalà, Magdalena Nowaczewska, Nicole Spare, Roberto Teggi, Maurizio Versino, Hsiangkuo Yuan, Ashley Zaleski-King, Michael Teixido, Frederick Godley

**Affiliations:** ^1^Department of Research, Association of Migraine Disorders, North Kingstown, RI, United States; ^2^Department of Otolaryngology-Head and Neck Surgery, Medical University of South Carolina, Charleston, SC, United States; ^3^Department of Otolaryngology-Head and Neck Surgery, The Johns Hopkins University School of Medicine, Baltimore, MD, United States; ^4^Department of Neurology and Neurotherapeutics, University of Texas Southwestern Medical Center, Dallas, TX, United States; ^5^Department of Otolaryngology-Head and Neck Surgery, University of California, Irvine, Irvine, CA, United States; ^6^Department of Otolaryngology, Keck School of Medicine, University of Southern California, Los Angeles, CA, United States; ^7^Department of Otolaryngology, University of Miami Miller School of Medicine, Miami, FL, United States; ^8^Jefferson Headache Center, Thomas Jefferson University, Philadelphia, PA, United States; ^9^Department of Otolaryngology-Head and Neck Surgery, Mass Eye & Ear and Harvard Medical School, Boston, MA, United States; ^10^Department of Otolaryngology-Head and Neck Surgery, Washington University School of Medicine, Saint Louis, MO, United States; ^11^Department of Research, Bayview Physicians Group, Chesapeake, VA, United States; ^12^Biomedical Engineering and Neuroscience, University of Rochester Medical Center, Rochester, NY, United States; ^13^Otolaryngology Unit, University of Siena, Siena, Italy; ^14^Department of Otolaryngology, Head and Neck Surgery, Laryngological Oncology, Nicolaus Copernicus University, Torun, Poland; ^15^Department of Otolaryngology, San Raffaele Scientific Hospital, Milan, Italy; ^16^Neurology and Stroke Unit, ASST Sette Laghi, Circolo Hospital, Varese, Italy; ^17^Department of Speech-Language Pathology & Audiology, Towson University, Towson, MD, United States

**Keywords:** vestibular migraine, chronic migraine (CM), trigemino-vascular pathway, vertigo-pathophysiology, vestibular disorders

## Abstract

Vestibular migraine (VM) is an increasingly recognized pathology yet remains as an underdiagnosed cause of vestibular disorders. While current diagnostic criteria are codified in the 2012 Barany Society document and included in the third edition of the international classification of headache disorders, the pathophysiology of this disorder is still elusive. The Association for Migraine Disorders hosted a multidisciplinary, international expert workshop in October 2020 and identified seven current care gaps that the scientific community needs to resolve, including a better understanding of the range of symptoms and phenotypes of VM, the lack of a diagnostic marker, a better understanding of pathophysiologic mechanisms, as well as the lack of clear recommendations for interventions (nonpharmacologic and pharmacologic) and finally, the need for specific outcome measures that will guide clinicians as well as research into the efficacy of interventions. The expert group issued several recommendations to address those areas including establishing a global VM registry, creating an improved diagnostic algorithm using available vestibular tests as well as others that are in development, conducting appropriate trials of high quality to validate current clinically available treatment and fostering collaborative efforts to elucidate the pathophysiologic mechanisms underlying VM, specifically the role of the trigemino-vascular pathways.

## Introduction

Vestibular migraine (VM) is a migraine disorder which presents with heterogeneous symptomatology and with several overlapping phenotypes. VM remains largely underdiagnosed and knowledge of its pathophysiology is limited (1). It is typically characterized by recurrent vestibular symptoms associated with migraine symptoms (headaches of a migraine quality and/or photophobia and phonophobia and/or visual aura) and has been considered among the most common causes of dizziness (2). Risk factors associated with VM include young age (below 40 years), female sex, a history of anxiety and depression and prior head trauma (3). Previously, other terms have been used for VM which include benign recurrent vertigo, migraine-associated dizziness, migraine-associated vertigo, migrainous vertigo, and migraine-related vestibulopathy.

Epidemiological data confirm that migraine-related syndromes that include a spectrum of vestibular symptoms are the most common cause of vertigo and dizziness in adults and children (4). While VM can develop at any age, it generally affects persons with a long-established history of migraine disease (5). It is diagnosed with an average delay of 8.4 years after the first onset of migraine (6).

Since the early development of VM as a distinct diagnosis in the 1980's, the estimated prevalence of VM in the general population is not precisely known because of the general underdiagnosis of this condition (7). According to Neuhauser et al. VM accounts for about 7% of patients seen in dizziness clinics and 9% of patients seen in migraine clinics (5). Other case series report that 16–34% of patients seen for dizziness in an otolaryngology clinic have a vestibular migraine (8–10). A national telephone interview survey in Germany by Neuhauser et al. estimated the 1-year and a lifetime prevalence of VM to be 0.89% and 1% in the adult general population (11). In a more recent study by Formeister et al. within the United States (U.S.), based on a population-based survey from the 2008 National Health Interview Survey (NHIS), the prevalence of VM in adults was estimated to be 2.7% (3). This is higher than the estimated prevalence of benign paroxysmal positional vertigo (BPPV) of 1.6% and that of Meniere's disease (MD) (0.2–0.5%) (12, 13).

On October 4, 2020, the Association of Migraine Disorders (AMD) hosted a half-a-day virtual workshop to discuss and identify research gaps in VM with the goal of identifying initiatives that would advance research in this field. The basis for convening this meeting was to evaluate the state of diagnosis, pathophysiology, and treatment of VM and furthermore, to answer some of the many challenging and unanswered questions related to this disease. Participants included experts from several medical specialties including otolaryngology, neurotology, neurology, audiology, and physical therapy. Based on the discussions at the workshop, this report highlights existing care gaps, supporting evidence and recommendations to address these gaps.

## Care Gap 1: Lack of Universally Accepted Range of VM Phenotypes and Unclear Spectrum of VM Disorders

In 2012, diagnostic criteria for VM were put forth by the International Headache Society (ICHD-3) and Bárány society which replaced the historical most accepted Neuheuser criteria (14, 15). Currently, there are multiple classification criteria for VM. The most restrictive is the ICHD-3 listed in Table 1 which acknowledges only definite VM. The Bárány society criteria makes provision for a separate category called probable vestibular migraine (Table 1) and in clinical practice, most patients who benefit from migraine therapy would fall in this latter category.

**Table 1:** Diagnostic criteria for VM as proposed by International Headache Society and Bárány Society.

**Vestibular Migraine:**
A. At least five episodes with vestibular symptoms lasting 5 min to 72 h.
B. Current or previous history of migraine with or without aura.
C. One or more migraine features with at least 50% of the vestibular episodes:
a. Headache with at least two of: one sided location, pulsating quality, moderate or severe pain intensity, aggravation by routine physical activity
b. Photophobia and phonophobia
c. Visual aura
D. Not better accounted for by another vestibular diagnosis or ICHD diagnosis
**Probable Vestibular Migraine:**
A. At least five episodes with vestibular symptoms lasting 5 min to 72 h.
B. Only one of the criteria B and C for vestibular migraine is fulfilled.
C. Not better accounted for by another vestibular diagnosis or ICHD diagnosis.

Current ICHD-3 and Bárány Society diagnostic criteria of vestibular migraine leave many endophenotypes/subtypes of VM unrecognized. For instance, dizziness *per se* is not exclusively a vestibular symptom qualifiable for VM diagnosis. To be considered, dizziness must be triggered by head motion and must be associated with nausea or other migraine symptoms. The current ICHD-3 symptoms that are symptoms qualifiable exclude certain dizziness phenotypes such as rocking and displacement in place seen in disorders such as Mal de debarquement (MDDS), Persistent Postural Perceptual Dizziness (PPPD), along with visual motion sensitivity. The rationale for specifying moderate or severe symptom severity is likely included to avoid duplicative diagnoses, or misattribution of dizziness symptoms (i.e., imbalance) when the primary symptom is headache. Evidence from current and future studies will help to elucidate the features of vestibular symptoms in VM and revise that point of the diagnostic criteria.

Epidemiological studies have documented the relationship between migraine headache and vertigo, suggesting an association between the two, yet the underlying neuronal pathophysiology is relatively unknown (16–18). Migraine headache typically predates the onset of vertigo with the mean duration of 8 years (6). Mean age of onset of headaches is 28 years old and mean age of dizziness onset is 49 years old. Patients with a synchronous or a combined presentation are younger (19). Additionally, the prevalence of vertigo is high in migraine patients and similarly, patients with dizziness often have a history of migraine (20). In the Migraine and Neck Pain Study, 30% of adult patients reported episodic vertigo anytime during the migraine attack with 16% having vertigo at the onset of headache, with 10% patients reporting episodic vertigo within 2 h before the headache and 3% patients experiencing episodic vertigo as a premonitory symptom up to 48 h before the headache. Nausea and vomiting were found to be associated with headaches and vertigo (19). Almost a third of these patients in the Migraine and Neck Pain Study could be diagnosed with VM or at least probable VM, according to the Bárány society criteria, supporting the association between migraine headache and vertigo (20). Symptoms such as cyclic vomiting, recurrent abdominal pain and atopy are also commonly observed in migraine patients (21). Considering there is an overlap of symptoms, both migraine and vestibular migraine may be part of a disease spectrum sharing an underlying pathophysiology.

**Recommendation 1:** (a) Establish a global VM patient registry that maintains uniform quality data and documents the natural history of vestibular symptoms in relation to migraine disease, (b) Refine the international, evidence-based diagnostic criteria for VM to account for the various symptoms reported by VM patients.

## Care Gap 2: VM Is Currently Underdiagnosed in the General Population and Multiple Associated Co-Morbidities are Underrecognized

Diagnosis for VM primarily relies on clinical history due to the lack of clinically available and reliable diagnostic laboratory tests. Since several epidemiological studies have supported the relationship between migraine and VM, the diagnosis of VM is very much dependent on the presence of migraine symptoms (22). Symptoms of dizziness and vertigo may be spontaneous, positional, visually induced, or head-motion induced, and are described in many ways by patients. Additionally, the timing of the attacks can also vary from minutes to days making the diagnosis of this disease very complex. For instance, as awareness of the relationship of migraine and vertigo has grown, many patients previously assigned a diagnosis of basilar migraine are now known to have VM. Basilar migraine is now better known as migraine with brainstem aura where vertigo and at least one other brainstem neurologic symptom (excluding motor weakness) must precede the headaches.

Several comorbidities are also known to be associated with VM including neurotologic conditions such as BPPV and MD. The symptoms of these conditions may overlap with those of VM symptoms and confuse the diagnosis of VM, especially when patients with other vestibular disorders may have superimposed, secondary migraine symptoms and not have VM as a primary cause of their symptoms (22). Psychiatric comorbidities, including anxiety, somatoform dizziness/chronic subjective dizziness may coexist with VM as well (23, 24). Furthermore, acknowledging that patients may have more than one vestibular diagnosis contributing to their symptoms is essential to providing accurate treatment and conducting accurate research (25).

There are suggestions that migraine mechanisms can affect the inner ear, that BPPV and MD can be complications of a migraine process, and that these conditions are potentially in pathophysiologic continuum with VM in which some patients manifest pure central disease, others manifest pure peripheral disease, and others have manifestations of both (26, 27). Patients with VM may exhibit tinnitus, aural pressure, and fluctuations in hearing. Hearing loss however does not tend to progress as it does in MD (28). Early on, MD patients may exhibit isolated episodic vertigo without otologic symptoms. Despite having a formal diagnostic criterion for VM and MD, it is becoming clearer that the diagnostic criteria for VM need to be refined and broadened (29). For instance, Frejo et al. define five endophenotypes for MD: idiopathic or classic, posttraumatic/delayed, autoimmune, familial or associated with migraine (30). The current ICHD-3 criteria acknowledge that there are patients with features of both disorders (with cochleovestibular symptoms of ear fullness/pressure, periodic or fluctuating tinnitus, fluctuating hearing loss in the setting of vestibular migraine or of a migraine disorder) and recommend treating the patients for MD especially if there is compelling audiometric confirmation. In addition to understanding the multiple phenotypic presentations of VM, patients with classifiable peripheral disease such as MD and recurrent BPPV responding to migraine management and with migraine features during attacks should potentially be considered to be on the same spectrum (31, 32). This broadening of recognized symptoms should be expanded to include patients with chronic rocking dizziness and patients with disorientation in space, as these symptoms are commonly reported by migraine patients. While MDDS is currently considered a separate entity, it is the hallmark condition causing rocking dizziness. Multiple studies show high prevalence of migraines especially in “spontaneous mal de debarquement” as well as high prevalence of new-onset headaches in patients with new onset MDDS (8, 33, 34).

**Recommendations 2: (a)** Enhance provider education, especially among practitioners who manage patients with dizziness, around VM and its potential subtypes including complexity of symptoms that may occur on a continuum, (b) Consider refining ICD coding for VM and possible subtypes as has been done for migraine headache.

## Care Gap 3: Lack of a Known Diagnostic Laboratory Testing or an Objective Marker Available for Clinicians

The current diagnostic criteria of VM are only clinical and based on the identification of the features outlined in the consensus of the International Headache and Bárány Societies in the ICHD-3 (14, 15). There is no pathognomonic clinical sign for vestibular migraine and there are no gold standard diagnostic tests for VM.

However, certain paraclinical tests are administered in the clinical setting. Auditory and vestibular testing has some value in the comprehensive evaluation of these patients. Classic audiometry can put in evidence a low frequency hearing loss suggestive of MD or concomitant MD. Other audiometric tests are not widely used in clinical practice. However, transient evoked otoacoustic emissions (TOAEs), distortion product otoacoustic emissions (DPOAEs), and auditory brainstem response (ABR) at high and low repetition rate frequencies show nonspecific abnormalities in nearly two thirds of patients with chronic migraine (35).

In addition, while vestibular testing cannot identify the presence of VM, it can put in evidence a concurrent dysfunction such as uncompensated vestibular hypofunction (due to peripheral or central mechanisms), comorbid BPPV as well as utricular/saccular dysfunction (35). Additionally, certain patterns can emerge on testing as well and help guide the diagnosis and/or the treatment plan (36). For instance, motion sensitive individuals tend to have longer time decay constants on step velocity rotary chair testing (37, 38). Some patients with vestibular migraines have caloric response asymmetry or may have oculomotor deficits or positional nystagmus with central features (39–41). While some studies points toward peripheral dysfunction in the setting of vestibular migraine, one should bear in mind that a caloric asymmetry can still indicate a separate vestibular disorder or a preexisting vestibular dysfunction which in itself may be a cause of migraines. Other observed patterns include the fact that VM patients are less likely to complete a full battery of vestibular tests and are more likely to develop an attack following testing (42, 43).

Vestibular-evoked Myogenic Potentials (VEMPs), a measure of saccular and utricular function, can separate in some instances VM from MD but the data is not clear-cut and there are no consistently reliable abnormalities (44–46). A recent systematic review did not support the role of cervical VEMP alone to aid in diagnosing VM (47). Rizk et al. (48) looked at the negative predictive value of the presence of an oVEMP and/or cVEMP response in patients presenting with a differential diagnosis of VM vs MD and showed that a present response in this specific clinical situation has a 93% negative predictive value. Subsequent studies suggested using VEMPs as part of a diagnostic algorithm.

On the other hand, current research is looking into other tools that could identify a patient with VM and differentiate them from other pathologies, namely MD. Tests of perceptual threshold, which are still not clinically available, show reduced dynamic tilt thresholds in vestibular migraine patients (49). The measurement of subjective visual vertical (SVV) and subjective visual horizontal (SVH) can serve as diagnostic indicators of dysfunction in spatial orientation (50). Abnormal SVV and SVH measurements occur in a significant number of individuals with VM, especially during head tilt where the brain is challenged to maintain spatial orientation (51, 52). The larger SVV errors in VM compared with healthy controls suggest abnormal sensory integration for spatial orientation in these patients (51). Thus, incorporating head-roll tilt during static and dynamic SVV conditions rather than in the traditional head upright position can produce more distinctive findings in migraine patients.

The functional head impulse test with and without optokinetic stimulation shows promise in identifying VM patients since it highlights dynamic visual dependence which is a hallmark manifestation of VM (53). The usefulness of this test in make a differential diagnosis with MD is to be evaluated (54).

Finally, there has been a quest to identify a readily accessible biomarker for VM. A recent study has identified a differential proinflammatory signature separately for patients with VM and MD where certain cytokines and chemokines were elevated. These preliminary studies suggest VM can be differentially diagnosed from healthy individuals and patients with MD using a small cytokine panel retrieved from a blood sample (IL-1β, IFNγ, CCL3, CCL22, and CXCL1) thereby improving the clinical management of VM (55).

**Recommendation 3:** (a) Create an improved diagnostic algorithm for VM using available vestibular function testing and consider incorporating perceptual threshold testing into clinical practice, (b) Pursue further research to identify a circulating biomarker to differentiate VM from MD.

## Care Gap 4: Pathophysiologic Mechanisms Explaining the Various Phenotypes and forms of VM (Episodic Vs. Chronic) as Well as Associated Symptoms (Spatial Disorientation, Hearing Loss, Vertigo) are Still Unclear

In general, very little is known about the pathophysiology of VM. Given the multiple potential interactions between the trigeminal and vestibular systems, it is likely that VM symptoms have a multifactorial etiopathophysiologic basis. Several possible mechanisms have been proposed based on the general understanding of migraine pathophysiology. Potential involvement and interactions between the trigemino-vascular system (TVS), nociceptive brain stem centers, thalamocortical network, and vestibular system have also been suggested to explain the underlying pathogenesis linking various VM symptoms (1).

Headaches are thought to result from an increased activity or sensitization within the TVS and nociceptive brainstem and thalamocortical centers. Animal models of chronic migraine suggest that migraine-mediated sensitization of the trigeminal nuclei, through its neural projections to vestibular nuclei, might affect the sensitivity of vestibular nuclei resulting in migraine-associated vestibular dysfunction (56). Certain neuropeptides such as calcitonin gene-related peptide (CGRP) and substance P cause neuroinflammation leading to pain or allodynia (central sensitization) in migraine patients. Some of these same neuropeptides (such as CGRP) are also expressed in the vestibular system and could be involved in VM pathophysiology (56). In fact, systemic CGRP injection has been shown to cause light-aversion (photosensitivity) in mice which can be blocked by CGRP antagonists or monoclonal antibodies (57). Furthermore, using a rat model of chronic migraine, Zhang et al. (56) demonstrated the possibility of sensitization of vestibular nucleus neurons to impair vestibular function after chronic migraine and were able to restore vestibular dysfunction in following anti-CGRP treatment (56). An increase in CGRP levels in both sensitized trigeminal and vestibular nuclei suggest that CGRP may have a critical role in the transformation to chronic sensitization. Therefore, just as CGRP enhances the abnormal pain sensitivity, it is possible that it might also increase the sensitivity of the balance system and can explain the spontaneous or sustained nature of VM (56).

Trigeminal innervation of the labyrinth vessels links the nociceptive trigeminal system to the vestibular system. Connections between brainstem vestibular nuclei and the structures that modulate trigeminal nociceptive inputs throughout the brainstem may play some part in VM. Neuroimaging techniques suggest an altered modulation in the integration and processing of vestibular and nociceptive information with the network from the vestibular sensors through the brainstem to the cortex. In this context, genetic variables can affect the excitatory–inhibitory balance involved in neural processing of sensory information, vestibular inputs, and pain (56).

Functional imaging data suggests the involvement of the multisensory vestibular thalamocortical networking in the pathophysiology of VM (1, 58, 59). Dizziness and spatial disorientation are thought to be caused by abnormal sensory modulation or integration within the thalamocortical network (1). Several findings indicate central dysfunction related to vestibular processing in patients such as perceptual dysfunction, abnormal spatial orientation during head tilt, elevated vestibulo-perceptual threshold after adaptation to visual motion (51, 60). These findings suggest that in general the perceptual functions of vestibular system are on a spectrum with VM on one end with low or distorted perception threshold and high level of symptoms (1). While, on the other end of the spectrum, there are those with high perceptual thresholds (such as ballet dancers) and low symptoms who show shorter durations of vestibular ocular response and perception of rotation (time constant) with whole body rotation (1, 61). Thus, an effective treatment strategy can be envisioned that could move patients toward the middle of the spectrum by increasing motion perception thresholds. Therefore, gaining insights into central processes that result in dizziness, vertigo, and spatial disorientation is an important step toward devising effective treatment strategies (1).

Altered activity of the vestibular system is thought to cause vestibular ocular dysfunction or vestibular hypersensitivity associated with migraine features (1). Also, a recent cross-sectional study suggested that cortical interactions between the visuo-vestibular system are abnormal in vestibular migraine patients (1). Results from peripheral vestibular testing in VM patients has shown inconsistent results indicating vestibular dysfunction and some of this dysfunction could be related to the modulation of inner ear function by migraine-related mechanisms. Furthermore, the sensory neuroepithelia within the vestibular sensory pathways are thought to be influenced by a diverse number of neuroactive substances that may act to enhance or inhibit the effect of the primary neurotransmitters such as glutamate and acetylcholine (62).

In addition, it is also hypothesized that integration of canal and otolith inputs for motion perception could be abnormal in VM, a function that is mediated by the cerebellar nodulus and uvula and/or possibly the thalamus (63). It was further hypothesized that elevated levels of CGRP during and between vertigo episodes could reduce Purkinje cell activity, thereby disinhibiting the neurons in the vestibular nuclei that receive their projections and contribute to the velocity storage network (64). Velocity storage has been linked to the synthesis of canal and otolith cues and motion sickness and aberrant control of this network could contribute to the vertigo and motion intolerance in VM (49, 63).

Finally, the vascular circulation of the inner ear receives innervation from the trigeminal nerve through the basilar artery and the anterior inferior cerebellar artery. Chemical and electrical stimulations of the trigeminal nerve may cause a significant increase in inner ear blood flow and neuroinflammation - changes in intravascular permeability and plasma protein extravasation into the inner ear (65). Vasospasm of the cochlear and/or vestibular branches of the internal auditory artery might explain sudden episodes of hearing loss and/or vertigo associated with migraine (49).

**Recommendation 4: (**a) Promote collaborative research (between laboratory scientists and clinicians) to study central and peripheral mechanisms of VM symptoms (b) Identify the effect of modulating the TVS, possibly by blocking CGRP, in the inner ear specifically and then understanding its effects on neuroepithelium in the auditory and vestibular system to provide some clues to VM pathophysiology.

## Care Gap 5: Lack of Understanding of the Biological Differences Underlying Gender Disparity in VM

While VM can occur at any age and between both sexes, it peaks in middle age and is 2–3 times more prevalent in women than in men (6, 66, 67). A common pattern/typical patient seen in a clinic is a woman with a history of classic or common migraines with improved symptoms following menopause but presenting with new-onset vestibular symptoms years after headaches have diminished (1, 68).

Changes in hormonal levels can be associated with episodes of vestibular migraine which can sometimes be observed in the perimenstrual period (69–71). Vestibular symptoms in women can also become more pronounced around the time of perimenopause. Dizziness is a common symptom in the late stage of menopausal transition and an unstable hormonal state is considered to be its trigger. While the role of sex hormones (estrogen and progesterone) in VM is not yet understood they are thought to influence various neurotransmitter systems in the brain that are associated with migraine activation such as serotonergic, glutaminergic, GABAergic, noradrenergic, and opioid systems (72).

**Recommendation 5:** Promote studies to understand possible associations between hormonal changes and vestibular symptoms across the lifespan.

## Care Gap 6: Lack of Appropriate Combination af Pharmacologic and Nonpharmacologic Measures to Treat Vestibular Migraine as Well as of an Appropriate Stepwise Management Algorithm

The current treatment approach of the VM patient is not well codified and is comprised of nonpharmacologic measures as well as medications prescribed (for the most part) in an off-label indication, and rehabilitative measures of uncertain potential. Most measures are based on empiric evidence or extrapolated from the treatment of typical migraine headaches.

Non-pharmacological measures such as diet, sleep hygiene and avoidance of triggers are recommended the same way they are for migraine headaches. Greater occipital nerve blocks and vagal nerve neuromodulators can be helpful as well (72, 73). Nutraceuticals have been proven to be effective in prevention of migraine headaches have not yet been studied thoroughly studied for VM.

The mainstay of the management of vestibular migraine is prophylactic medication when VM attacks are frequent and severe. Migraine headache prophylactic medications have been traditionally used to treat VM and observational studies with these medications have shown benefits (74). Pharmacological drugs for VM have been used for acute treatment or as preventative treatments and are classified into two groups based on their mechanism of action. One class of drugs act on the neurotransmitters and their receptors. The second class of drugs act on voltage-gated channels (75). The details of the current drugs used for treatment of VM along with their daily dosage and common side effects have been reviewed extensively before (74, 76, 77). Typically, the choice of medication for treating VM is guided by its side effect profile and the comorbidities of patients. Interestingly, these treatment recommendations have been based on observational studies, physicians' experiences, familiarity with the medications, patient comorbidities and side effects of various interventions (74, 78).

Treatment trials for VM are rare and only now starting to emerge. Previously, only two randomized controlled trials were conducted with triptans (rizatriptan and zolmitriptan) specifically for acute attacks to VM and these provided limited evidence for treating VM (79). A recent systematic review and meta-analysis of 13 studies assessed the efficacy of various therapies for prevention of VM. Treatment options analyzed included antiepileptic drugs, calcium channel blockers, tricyclic antidepressants, beta blockers, selective serotonin reuptake inhibitors (SSRIs), serotonin and norepinephrine reuptake inhibitors (SNRIs) and vestibular rehabilitation (78). All these treatments showed improvement in measured outcomes, but an established preferred treatment could not be determined due to the heterogeneity of the VM symptoms and the lack of standardized reporting on outcomes (8, 78, 80).

Experiencing vertigo can produce anxiety, therefore treating or managing comorbidities, particularly mood disorders, can help with vestibular symptoms (81). Tricyclic antidepressants such as amitriptyline or nortriptyline, or SSRIs and benzodiazepines such as clonazepam have been recommended (82). SNRI have also been suggested as clinically safe and effective for migraine as well as VM prophylaxis but were considered inferior in response to other drugs (83).

CGRP monoclonal antibodies are novel therapies that have been approved for either prevention or treatment of migraine. Clinically we can assume that by controlling migraines in patients with CGRP monoclonal antibodies, we may be able to reduce or eliminate symptoms of VM. There is currently an active single center pilot study with randomized double-blinded placebo-controlled trial comparing the efficacy and safety of galcanezumab to placebo in the treatment of vestibular migraine (https://clinicaltrials.gov/ct2/show/NCT04417361). Preliminary data from another monoclonal antibody eptinezumab (NCT04152083) for prevention of chronic migraine were positive, but it is yet to be approved by the FDA.

Serotonin receptor 5-HT_IF_ is believed to regulate the release of CGRP from vestibular nuclei (84). In 2019, FDA approved 5-HT_IF_ receptor agonist lasmiditan for treatment of acute migraine (85). Considering Lasmiditan was effective in treating headache and photophobia, it may have the potential to serve as a prophylactic treatment for VM.

Vestibular rehabilitation and natural activities that can enhance spatial perception and body coordination, such as ping-pong and dancing can be helpful to alleviate symptoms in VM patients. Vestibular rehabilitation can be useful when there is associated vestibular dysfunction or comorbid BPPV but also when patients develop maladaptive strategies that lead to visual dependence or loss of confidence in balance (82). More studies are warranted to truly understand the benefits of vestibular rehabilitation and other rehabilitative strategies in the VM patients (86). Rehabilitation should be conducted by an experienced therapist who will be able to slowly and incrementally increase the dose of the exercises to avoid causing significant symptoms since these patients are generally very motion sensitive.

Finally, current treatment strategies are focused on reducing the burden of vestibular and headache symptom burden. Often overlooked symptoms such as “brain fog,” “foggy headed,” “head in a cloud” and “difficulty thinking clearly” correlate with worse scores on cognitive scales and are best classified as forms of cognitive dysfunction. Cognitive dysfunction may be a cause of ongoing disability in VM patients. There is currently no clear intervention that would help address this specific symptomatology although cognitive therapy or other rehabilitative measures could be explored (48, 87).

**Recommendation:** (a) Promote novel and evidence-based approaches for VM treatment, (b) Develop and publish guidelines on conducting and reporting randomized controlled trials of acute and preventative treatment of VM modeled on available guidelines developed by the ICHD for migraine cephalgia trials (88), (c) Conduct randomized control trials to study pharmaceutical and non-pharmaceutical approaches for VM treatment.

## Care Gap 7: Need for Better Patient Reported Outcome Measure (Prom) to Quantify the Impact of VM or Any Intervention on the Patient's Quality of Life

Vestibular disorders are a major cause of absenteeism and loss of productivity at work (89). In addition, vestibular disorders in general and VM in particular have been associated with sleep disturbances, psychiatric comorbidities and cognitive dysfunction. Furthermore, chronic migraine, by itself, is considered the second most prevalent cause of disability worldwide (90). Given the prevalence of these disorders and their impact on patients, it can be inferred that vestibular disorders, particularly VM can have a major impact on a patients' quality of life.

The current available tools such as the dizziness handicap inventory, and PROMs are not disease specific and do not capture the extent of the condition's impact (91–93). There is also a need for better PROMs for research study design (critical to improving success in NIH funded projects). Efforts are being made in that area with Sharon et al. recently describing a potential new instrument VM-PATHI (94). In addition to being able to gauge the severity of the condition, in the absence of an objective marker for diagnosis, we currently also rely on patients' reports to gauge the response to any intervention. PROMs are used in most clinical trials as outcome measures. To this end, there is a need to properly define appropriate outcome measures before designing and studying treatment interventions.

**Recommendation 7:** Create and validate a VM disease-specific PROM instrument using psychometrically valid methods.

## Concluding Statement

We have identified seven broad care gaps in our understanding and management of vestibular migraine. There is a lack of definition of endophenotypes of VM and underdiagnosis of subjects with atypical symptoms or who do not fit the ICHD-3 criteria strictly. The absence of an objective diagnostic marker contributes to those gaps as well as the insufficient knowledge of pathogenesis including hormonal influence. Furthermore, there is insufficient evidence of best treatment algorithms and treatment is often initiated based on comorbidities and side effect profile without clear evidence for a stepwise approach. Finally, there is a need to develop an instrument that can capture all the dimensions of the quality of life affected by the VM condition; this will help gauge the severity of the condition but also may be used as an outcome measure of interventions. The recommendations proposed by this group to address the care gaps, listed together in Table 2, broadly fall into three categories, namely establishing a VM registry, advancing clinical research on VM and providing education. Establishing a registry can allow for creating a treatment algorithm, a diagnostic algorithm as well as retrieval of high-quality data provided an ICD code is created. Advancing clinical research is critical as many knowledge gaps remain to this day. Encouraging and fostering collaborations with basic researchers can promote an understanding of the underlying pathophysiological mechanisms of VM and its connection to chronic migraine and other comorbidities. Although treatments are available, there is a critical need for evidence-based approaches to treating and managing VM. More clinical trials for novel treatments are needed with appropriate outcome measures along with comparative efficacy studies for existing treatments. Finally, broader education and awareness on VM, its endophenotypes and its relation to comorbidities is clearly needed for clinicians who treat patients for dizziness.

**Table 2:** List of care gaps and recommendations for vestibular migraine proposed by the expert panel.

**Care Gaps**	**Recommendations**
1. Lack of universally accepted range of VM phenotypes and unclear spectrum of VM disorders.	(1a) Establish a global VM patient registry that maintains uniform quality data and documents the natural history of vestibular symptoms in relation to migraine disease. (1b) Refine the international, evidence-based diagnostic criteria for VM to account for the various symptoms reported by VM patients.
2. VM is currently underdiagnosed in the general population and multiple associated co-morbidities are underrecognized.	(2a) Enhance provider education, especially among practitioners who manage patients with dizziness, around VM and its potential subtypes including complexity of symptoms that may occur on a continuum (2b) Consider refining ICD coding for VM and possible subtypes as has been done for migraine headache.
3. Lack of a known diagnostic laboratory testing or an objective marker available for clinicians.	(3a) Create an improved diagnostic algorithm for VM using available vestibular function testing and consider incorporating perceptual threshold testing into clinical practice. (3b) Pursue further research to identify a circulating biomarker to differentiate VM from MD.
4. Pathophysiologic mechanisms explaining the various phenotypes and forms of VM (episodic versus chronic) as well as associated symptoms (spatial disorientation, hearing loss, vertigo) are still unclear.	(4a) Promote collaborative research (between laboratory scientists and clinicians) to study central and peripheral mechanisms of VM symptoms. (4b) Identify the effect of modulating the TVS, possibly by blocking CGRP, in the inner ear specifically and then understanding its effects on neuroepithelium in the auditory and vestibular system to provide some clues to VM pathophysiology.
5. Lack of understanding of the biological differences underlying gender disparity in VM.	5. Promote studies to understand possible associations between hormonal changes and vestibular symptoms across the lifespan.
6. Lack of appropriate combination of pharmacologic and nonpharmacologic measures to treat vestibular migraine as well as of an appropriate stepwise management algorithm.	(6a) Promote novel and evidence-based approaches for VM treatment. (6b) Develop and publish guidelines on conducting and reporting randomized controlled trials of acute and preventative treatment of VM modeled on available guidelines developed by the ICHD for migraine cephalgia trials. (6c) Conduct randomized control trials to study pharmaceutical and non-pharmaceutical approaches for VM treatment.
7. Need for better Patient Reported Outcome Measure (PROM) to quantify the impact of VM or any intervention on the patient's quality of life.	7. Create and validate a VM disease-specific PROM instrument using psychometrically valid methods

## Author Contributions

AK, SB, MT, and FG provided critical review and input on the initial drafts. All authors contributed to manuscript revision, read, and approved the submitted version.

## Funding

The funds used for publication fees were received from the Association of Migraine Disorders (AMD). AMD is a non-profit organization and strives to expand the understanding of migraine by supporting research, education, and awareness.

## Conflict of Interest

NS serves on the speaker's bureau for Abbvie and Amgen as well as an educational consultant to Amgen. FG serves on the speakers bureau for BioHaven and Amgen and as a consultant for Allergan. HD has a patent pending: “Combination therapy for the treatment of vertigo.” The remaining authors declare that the research was conducted in the absence of any commercial or financial relationships that could be construed as a potential conflict of interest.

## Publisher's Note

All claims expressed in this article are solely those of the authors and do not necessarily represent those of their affiliated organizations, or those of the publisher, the editors and the reviewers. Any product that may be evaluated in this article, or claim that may be made by its manufacturer, is not guaranteed or endorsed by the publisher.

## Abbreviations

VM: vestibular migraine
MC: migraine cephalgia
BPPV: benign paroxysmal positional vertigo
MD: Meniere's Disease
MDDS: Mal de debarquement syndrome
PPPD: persistent postural perceptual dizziness
TOAEs: transient evoked otoacoustic emissions
DPOAEs: distortion product otoacoustic emissions
ABR: auditory brainstem response
VEMP: vestibular-evoked myogenic potentials
SVV: subjective visual vertical
SVH: subjective visual horizontal
TVS: trigemino-vascular system
CGRP: calcitonin gene-related peptide
SSRIs: selective serotonin reuptake inhibitors
SNRIs: serotonin and norepinephrine reuptake inhibitors
PROMs: patient reported outcomes measures.
